# Physicochemical Characterization of a Strontium Silicate-Based Root Canal Sealer Compared with Two Conventional Sealers

**DOI:** 10.3390/jfb17040168

**Published:** 2026-04-01

**Authors:** Loai Alsofi

**Affiliations:** Department of Endodontics, Faculty of Dentistry, King Abdulaziz University, P.O. Box 80209, Jeddah 21589, Saudi Arabia; lalsofi@kau.edu.sa; Tel.: +966-555-318-481

**Keywords:** root canal obturation, endodontic sealers, calcium silicate, strontium, net mass change (apparent solubility behavior), scanning electron microscopy

## Abstract

Objectives: To evaluate the physicochemical properties of a novel strontium silicate-based root canal sealer (C-Root SP) in comparison with a calcium silicate-based sealer (TotalFill BC) and an epoxy resin-based sealer (AH Plus). Methods: Setting time, net mass change (apparent solubility behavior), pH changes, and surface characteristics were assessed based on ISO 6876 and ANSI/ADA Specification No. 57, with minor methodological modifications. Net mass change and pH were evaluated over 28 days. Surface morphology and elemental composition were analyzed after dry and aqueous aging in deionized water using scanning electron microscopy coupled with energy-dispersive X-ray spectroscopy. Data were analyzed using one-way and repeated-measures ANOVA with Tukey’s post hoc test (α = 0.05). Results: AH Plus exhibited the longest initial and final setting times (10.93 ± 0.65 h and 37.33 ± 0.13 h), whereas TotalFill BC showed the shortest (7.98 ± 0.32 h and 30.18 ± 0.20 h); C-Root SP demonstrated intermediate values (9.35 ± 0.38 h and 32.75 ± 0.57 h) (*p* < 0.001). C-Root SP exhibited positive net mass change values (indicative of net mass loss), ranging from 5.32 ± 4.72% at 24 h to 6.83 ± 5.55% at 28 days, significantly higher than AH Plus and TotalFill BC (*p* < 0.001), which showed negative values indicative of apparent mass gain. All sealers demonstrated alkaline conditions, with C-Root SP maintaining the highest apparent pH values throughout the evaluation period (*p* < 0.001). Surface and compositional changes were observed in the bioceramic sealers following aqueous aging, with increased detectable strontium content in C-Root SP. Conclusions: C-Root SP exhibited physicochemical behavior consistent with a strontium-modified calcium silicate-based sealer, characterized by hydration-driven hydroxyl ion release resulting in apparent alkalinity and ion exchange-associated behavior, and dynamic surface changes consistent with those reported for bioceramic materials. Clinical Significance: Strontium incorporation may influence hydration-mediated physicochemical behavior; however, further in vitro and in vivo studies are required to determine its clinical relevance.

## 1. Introduction

Long-term success of root canal treatment depends on adequate cleaning and shaping of the root canal system and the subsequent establishment of a three-dimensional (3D) bacteria-tight obturation [1,2]. Gutta-percha alone is insufficient to generate an effective 3D obturation because it lacks adhesion to dentinal walls. Therefore, endodontic sealers play a critical role in achieving a fluid-tight and bacteria-tight seal, which directly influences the prognosis of endodontic therapy [2]. Over the years, various sealers with different chemical compositions have been introduced to improve obturation quality and enhance long-term sealing performance [3].

Epoxy resin-based sealers such as AH Plus are considered the gold standard due to their favorable handling properties, low solubility, dimensional stability, and strong adhesion to canal walls [4,5,6]. AH Plus is a two-paste epoxy resin-based sealer composed primarily of bisphenol-A and bisphenol-F epoxy resins combined with amine hardeners, which polymerize through an addition reaction rather than a hydration-based mechanism [7]. This resin-based chemistry provides high flowability and promotes micromechanical interlocking with root canal dentin, and additionally enables covalent bonding between epoxy resin groups and the amino groups of dentinal collagen, thereby contributing to its superior sealing ability and long-term dimensional stability [8,9,10]. However, because AH Plus does not release calcium or hydroxyl ions and does not undergo hydration reactions, it lacks intrinsic bioactivity and shows limited ability to induce mineralized tissue formation or interfacial biomineralization [11].

In addition, several studies have reported that epoxy resin-based sealers may elicit transient inflammatory or cytotoxic responses, particularly during their unset or early setting phases, due to the release of unreacted resin components or amine derivatives [12,13,14]. These biological limitations, together with the absence of sustained ion release and alkalinizing capacity, may compromise long-term antimicrobial effects and the complete prevention of bacterial leakage, as previously reported [15]. Such drawbacks highlight the need for alternative sealers that combine acceptable physicochemical stability with enhanced biological activity.

In contrast, bioceramic sealers, particularly those based on tricalcium silicate, have gained attention for their osteogenic potential, biocompatibility, antimicrobial properties, and ability to induce biomineralization through hydration-driven ionic release and surface mineralization processes [4,16,17,18]. TotalFill BC, a widely used premixed hydrophilic calcium silicate-based formulation, is composed of tri- and di-calcium silicates, calcium phosphate, calcium hydroxide, and zirconium oxide, and is free of aluminum. It has been used as a reference bioceramic sealer in comparative studies [19,20,21].

Recent investigations have characterized the microstructural and chemical behavior of calcium silicate-based sealers using SEM-EDX and micro-CT analysis, confirming their Ca- and Si-dominant composition and favorable adaptation to dentin walls [22]. Such materials are known to form an apatite-like layer when exposed to physiological fluids, a feature closely associated with their bioactivity and biological sealing ability [16,17]. However, despite these advances, currently available bioceramic sealers rely exclusively on calcium-based matrices and lack elemental or compositional modifications that may further enhance ion release, surface reactivity, and cellular responses [17,23].

Strontium (Sr), a divalent cation chemically similar to calcium, has been shown to play a beneficial role in bone metabolism by promoting bone formation and inhibiting resorption [24]. Beyond its effects on bone turnover, strontium ions have been reported to modulate cellular behavior by enhancing osteogenic differentiation, reducing osteoclast activity, and exerting immunomodulatory effects that support tissue regeneration [25,26]. When incorporated into calcium silicate systems, strontium has been reported to enhance radiopacity, hydration kinetics, bioactivity, and mechanical properties [27], as well as to promote apatite formation and favorable ionic release profiles when exposed to physiological environments [28,29].

Recently, a novel strontium silicate-based root canal sealer (C-Root SP; Beijing Dental Medical Devices Co., Ltd., Beijing, China) has been introduced. It is a hydrophilic, aluminum-free material composed of strontium silicate, calcium phosphate, calcium hydroxide, zirconia, and potassium dihydrogen phosphate. Strontium silicate-based bioceramic formulations have been shown to exhibit improved cytocompatibility, enhanced cell migration, and osteogenic potential compared with conventional calcium silicate sealers, particularly at appropriate ion concentrations [30,31]. Because even small changes in sealer chemistry can significantly influence its physical and biological behavior, evaluating this new formulation is essential. In vitro physicochemical assessments are commonly employed as initial screening approaches to characterize dental materials and their interaction with aqueous environments prior to more advanced biological or clinical investigations.

Therefore, this study aimed to evaluate the physicochemical properties of a newly introduced strontium silicate-based sealer (C-Root SP) and to compare its performance with that of a representative conventional bioceramic sealer (TotalFill BC) and an epoxy resin-based sealer (AH Plus). The null hypothesis was that there would be no significant differences among the tested sealers in terms of setting time, net mass change (apparent solubility behavior), pH, and surface characteristics.

## 2. Materials and Methods

### 2.1. Materials Used

The sealers evaluated in this study included a novel strontium silicate-based sealer (C-Root SP, Beijing Dental Medical Devices Co., Ltd., Beijing, China), a bioceramic sealer (TotalFill BC, Brasseler, Savannah, GA, USA), and an epoxy resin-based sealer used as the gold standard control (AH Plus, Dentsply DeTrey, Konstanz, Germany). These three products were selected to represent distinct material classes currently used in endodontic obturation. All materials were obtained from manufacturer-sealed packages and used according to the manufacturers’ instructions. To minimize variability, all specimens within each group were prepared from the same batch; however, specific lot numbers were not recorded.

### 2.2. Sample Size

Sample size was determined based on previously published physicochemical studies of endodontic sealers employing comparable experimental designs and sample sizes (n = 5–10 per group) [11,20,32]. A standardized sample size of ten specimens per group (n = 10) was selected for all quantitative analyses (setting time, net mass change, and pH) to ensure adequate statistical power and comparability with prior investigations.

For SEM/EDX analysis, five specimens per group (n = 5) were evaluated, consistent with established microstructural methodologies, where limited specimen numbers combined with multiple analyzed regions are considered acceptable due to the localized and descriptive nature of SEM-based surface characterization and EDX analysis [33,34].

Post hoc power analysis based on observed effect sizes demonstrated statistical power exceeding 0.80 for the primary outcomes, confirming the adequacy of the selected sample sizes. Power analysis was performed using G*Power software (Version 3.1, Heinrich Heine University, Düsseldorf, Germany).

### 2.3. Setting Time

The setting time was determined using a Vicat needle apparatus (Jin-Ching-Her, Taiwan) based on ISO 6876:2025 specifications [35], with minor methodological modifications. Polyethylene molds (10 mm diameter × 3 mm height) were filled with premixed sealers (C-Root SP and TotalFill BC) or freshly mixed AH Plus (n = 10). Although ISO 6876 specifies a specimen thickness of 2 mm, a height of 3 mm was used to ensure sufficient bulk material and to minimize potential edge effects and premature surface drying, while maintaining the standardized 10 mm diameter [36]. This modification was introduced to improve handling stability and does not affect the comparative nature of the measurements. For freshly mixed AH Plus, time measurement began at the start of mixing. For premixed sealers, timing commenced immediately after extrusion into the mold. Specimens were maintained at 23 ± 1 °C and 50 ± 5% relative humidity throughout testing.

A Vicat needle (100 g load, flat-ended tip) (Jin-Ching-Her Co., Ltd., Taipei, Taiwan) was applied vertically to the specimen surface. Indentation testing began 5 min after mixing. Measurements were performed at 10 min intervals during the early setting phase to improve the detection accuracy of the initial setting time. As the setting progressed, intervals were extended to 20–30 min to minimize repeated surface disturbance while allowing precise identification of the final set [35]. The initial setting time was defined as the elapsed time from the start of mixing (or extrusion for premixed materials) until the indenter needle failed to produce a complete circular indentation on the material surface, indicating the onset of hardening.

The final setting time was recorded as the time at which the indenter no longer produced any visible indentation under the specified load, indicating that the material had achieved sufficient rigidity.

All measurements were conducted under standardized environmental conditions to minimize experimental variability.

### 2.4. Net Mass Change (Apparent Solubility Behavior)

Following determination of the final setting time, the same specimens were stored at 37 °C and 100% relative humidity for 7 days to ensure complete maturation, in accordance with ADA Specification No. 57 [37]. After this period, the initial dry mass (W_0_) of each specimen was measured using an analytical balance (Model ZSA210, Scientech Inc., Boulder, CO, USA). Each specimen was then immersed individually in 10 mL of deionized water (DIW) and stored at 37 °C for evaluation periods of 1, 7, 14, 21, and 28 days.

At each time interval, specimens were removed from the storage solution, gently blotted to remove excess surface moisture, air-dried overnight under controlled laboratory conditions, and reweighed to obtain the corresponding mass values (W_t1_, W_t7_, W_t14_, W_t21_, and W_t28_). Net mass change (expressed as percentage mass variation) was calculated using the following formula [38]:

$$Net\;mass\;change\;\%=\frac{W_{0}-W_{t}}{W_{0}}\times 100$$

Positive values indicate net mass loss, whereas negative values indicate apparent mass gain, which may be attributed to water sorption and/or surface precipitation rather than true material dissolution.

### 2.5. pH Changes

The pH of the storage solution used for the net mass change test was recorded at 1, 7, 14, 21, and 28 days using a calibrated pH meter (JENWAY 3510, Bibby Scientific Ltd., Stone, Staffs, UK). Calibration was performed at 25 °C using standard buffer solutions (pH 4.0 and 7.0) prior to each measurement session.

Importantly, the same deionized water was retained throughout the experimental period to allow cumulative assessment of ion release and its effect on solution pH over time within a closed system. No replacement of the storage medium was performed between time points. All measurements were performed in triplicate to ensure reliability.

It should be noted that pH measurements in highly alkaline solutions may exceed the theoretical limit of 14 due to limitations in electrode calibration and measurement accuracy at elevated ionic concentrations. Therefore, values above 14 were interpreted as indicative of strongly alkaline conditions rather than absolute pH values.

### 2.6. Surface Structure

For scanning electron microscopy (SEM) and energy-dispersive X-ray spectroscopy (EDX) analysis, five disc-shaped specimens per group (n = 5) (10 mm in diameter × 3 mm in thickness) were prepared using polypropylene (PP) molds (custom-made laboratory molds). Specimens were allocated to two aging conditions: dry storage at 37 °C for 28 days or immersion in deionized water (DIW) at 37 °C for 28 days. The tested sealers were handled according to the manufacturers’ instructions, and all specimens within each group were prepared from the same batch to minimize variability.

Following the aging period, immersed specimens were removed from DIW, gently rinsed with distilled water to remove loosely adherent surface residues, and air-dried under controlled laboratory conditions until a constant mass was achieved prior to examination.

Specimens were mounted on aluminum SEM stubs (Ted Pella Inc., Redding, CA, USA) using conductive carbon adhesive tape (Agar Scientific Ltd., Stansted, UK) and sputter-coated with a gold layer (~10 nm) using a sputter coater (Quorum Q150R ES, Quorum Technologies Ltd., Lewes, UK) for 60 s to improve surface conductivity and minimize charging.

Surface morphology was examined using a scanning electron microscope (SEM; JSM-6510LV, JEOL Ltd., Tokyo, Japan) operated at an accelerating voltage of 15 kV, with magnifications up to 7500×, to evaluate surface texture, porosity, and morphological changes associated with hydration and aging.

For each specimen, five regions of interest (ROIs) were randomly selected from representative areas of the surface at standardized magnification to minimize selection bias and ensure reproducibility. Care was taken to avoid edge artifacts and surface defects not representative of the bulk material.

EDX analysis (Oxford Instruments, Abingdon, UK) was performed in conjunction with SEM to determine surface elemental composition. Semi-quantitative elemental analysis was conducted on the same five ROIs per specimen, and the mean values were calculated to obtain a single representative value per specimen for statistical analysis.

It should be noted that EDX provides surface-sensitive, semi-quantitative elemental analysis and does not necessarily reflect the bulk composition of the material.

### 2.7. Statistical Analysis

Statistical analyses were performed using SPSS software (Version 16.0; SPSS Inc., Chicago, IL, USA). Data distribution was assessed using the Shapiro–Wilk test prior to inferential analysis, and all datasets satisfied normality assumptions (*p* > 0.05). Homogeneity of variances was confirmed using Levene’s test.

Accordingly, parametric tests were applied. Between-group comparisons for setting time, net mass change, and pH were performed using one-way analysis of variance (ANOVA) followed by Tukey’s post hoc test. Within-group changes over time were analyzed using repeated-measures ANOVA.

For SEM/EDX analysis, five regions of interest were examined per specimen and averaged to obtain a single representative value per specimen (n = 5 per group), ensuring independence of observations.

Effect sizes were calculated using partial eta squared (η^2^) to quantify the magnitude of differences between groups. Post hoc power analysis based on observed effect sizes confirmed statistical power exceeding 0.80 for the primary comparisons.

To explore potential associations between net mass change and pH, Pearson correlation coefficients and simple linear regression analyses were performed using paired measurements obtained at corresponding time points up to 28 days. These analyses were exploratory and intended to describe material-specific relationships rather than infer causality.

The level of statistical significance was set at *p* < 0.05 for all analyses.

## 3. Results

### 3.1. Setting Time

**Figure 1:**
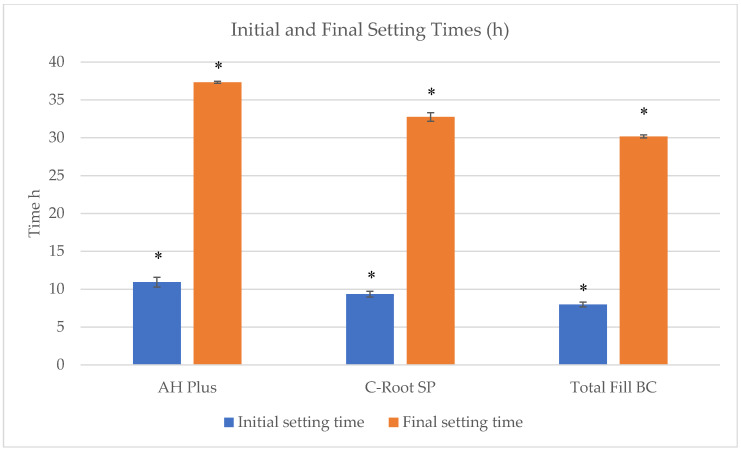
Comparison of the initial and final setting times of the tested root canal sealers (AH Plus, C-Root SP, and TotalFill BC). Data are presented as mean values (h). Final setting times were substantially higher than initial setting times for all groups, with AH Plus showing the longest setting time and TotalFill BC the shortest. “*” indicates a statistically significant difference between groups (*p* < 0.05).

The mean initial and final setting times of the tested sealers are presented in Table 1 and Figure 1. AH Plus exhibited the longest initial and final setting times (10.93 ± 0.65 h and 37.33 ± 0.13 h, respectively), whereas TotalFill BC demonstrated the shortest values (7.98 ± 0.32 h initial and 30.18 ± 0.20 h final). C-Root SP showed intermediate initial and final setting times (9.35 ± 0.38 h and 32.75 ± 0.57 h, respectively).

One-way ANOVA revealed statistically significant differences among the three sealers for both initial and final setting times (*p* < 0.001). Tukey’s post hoc analysis confirmed that all pairwise comparisons were statistically significant (*p* < 0.05).

### 3.2. Net Mass Change (Apparent Solubility Behavior)

Net mass change (apparent solubility behavior) values for all tested sealers at each time interval are presented in Table 2. C-Root SP exhibited positive net mass change values (indicative of net mass loss) across all evaluation periods, ranging from 5.32 ± 4.72% at 24 h to 6.83 ± 5.55% at 28 days. In contrast, TotalFill BC and AH Plus demonstrated negative values (indicative of apparent net mass gain) at all time points. TotalFill BC showed values ranging from −4.28 ± 0.64% at 24 h to −2.69 ± 0.54% at 28 days, whereas AH Plus exhibited minimal changes, ranging from −0.15 ± 0.21% at 24 h to −0.60 ± 0.23% at 28 days.

One-way ANOVA revealed statistically significant differences among the three sealers at each evaluated time point (*p* < 0.001). Tukey’s post hoc analysis confirmed that C-Root SP exhibited significantly higher net mass change values than both AH Plus and TotalFill BC at all intervals (*p* < 0.05). Significant differences were also observed between AH Plus and TotalFill BC at each time point (*p* < 0.05).

C-Root SP demonstrated greater variability in net mass change values compared with the other materials, as reflected by higher standard deviations (ranging from ±4.27 to ±6.38), suggesting heterogeneous mass change behavior among specimens. In contrast, AH Plus and TotalFill BC exhibited relatively low variability (generally ≤±0.64), indicating more consistent mass change patterns over time.

Repeated-measures ANOVA demonstrated no statistically significant changes in net mass change over time for AH Plus (*p* = 0.31) or C-Root SP (*p* = 0.30). In contrast, TotalFill BC showed a statistically significant overall time effect (*p* = 0.005); however, Tukey-adjusted pairwise comparisons did not reveal significant differences between individual time intervals.

### 3.3. pH Measurements

**Figure 2:**
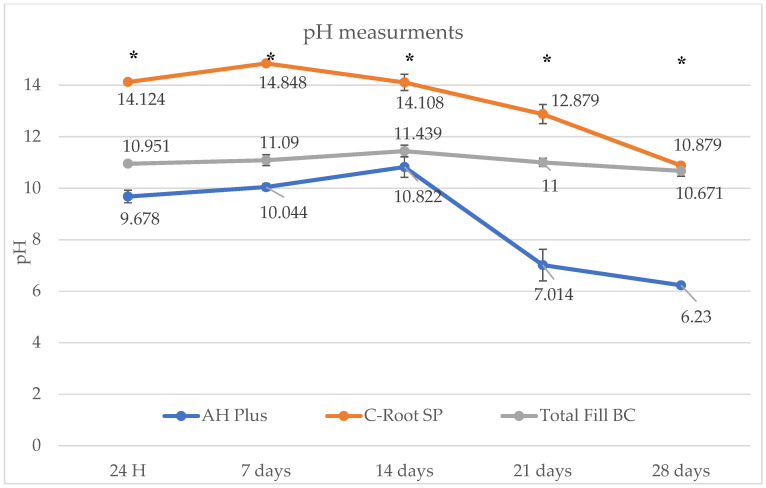
pH changes of AH Plus, C-Root SP, and TotalFill BC over 28 days. Data are presented as mean ± standard deviation. C-Root SP exhibited the highest apparent pH values at all time points, whereas AH Plus showed the lowest pH values. TotalFill BC demonstrated moderate and relatively stable apparent alkalinity over time. Asterisks (*) indicate statistically significant differences among sealers at each time point (*p* < 0.05). Because the same storage medium was retained throughout the experiment, the values reflect cumulative alkalinization rather than independent time-point measurements. Values exceeding pH 14 should be interpreted as indicative of strongly alkaline conditions, reflecting limitations of pH measurement under highly alkaline environments rather than absolute pH values.

The mean pH values of the tested sealers at each evaluation period are presented in Table 3 and illustrated in Figure 2. Statistically significant differences among materials were observed at all time points (one-way ANOVA, *p* < 0.001).

C-Root SP demonstrated the highest pH values at all evaluation intervals, with peak apparent alkalinity observed at 7 days (14.85 ± 0.02), followed by a gradual decline over time, while remaining within the alkaline range at 28 days (10.88 ± 0.10). TotalFill BC exhibited intermediate pH values, showing relatively stable apparent alkalinity throughout the experimental period (10.67–11.44). In contrast, AH Plus demonstrated the lowest pH values, with a progressive decrease over time, reaching its minimum at 28 days (6.23 ± 0.09).

It should be noted that the same storage medium was retained throughout the experiment; therefore, the reported pH values reflect cumulative alkalinization rather than discrete time-point measurements under refreshed conditions.

Repeated-measures ANOVA revealed statistically significant changes in pH over time for all tested sealers (*p* < 0.001). Within-group comparisons showed significant differences between specific time intervals for each material, as indicated by uppercase superscripts in Table 3.

Pearson correlation and simple linear regression analyses were performed to explore potential associations between net mass change and pH values over the 28-day evaluation period. Material-specific relationships were observed (Table 4).

C-Root SP demonstrated a strong positive correlation between net mass change and pH (r = 0.86, R^2^ = 0.75). In contrast, TotalFill BC and AH Plus exhibited negative correlations (r = −0.58, R^2^ = 0.34 and r = −0.79, R^2^ = 0.62, respectively), indicating inverse relationships. These findings should be interpreted as descriptive associations rather than evidence of direct causative relationships.

Correlation and regression analyses were based on paired net mass change and pH measurements obtained at corresponding time points. The Pearson correlation coefficient (r) indicates the strength and direction of the association, while the coefficient of determination (R^2^) reflects the proportion of variance in net mass change explained by pH.

### 3.4. EDX Analysis

**Figure 3:**
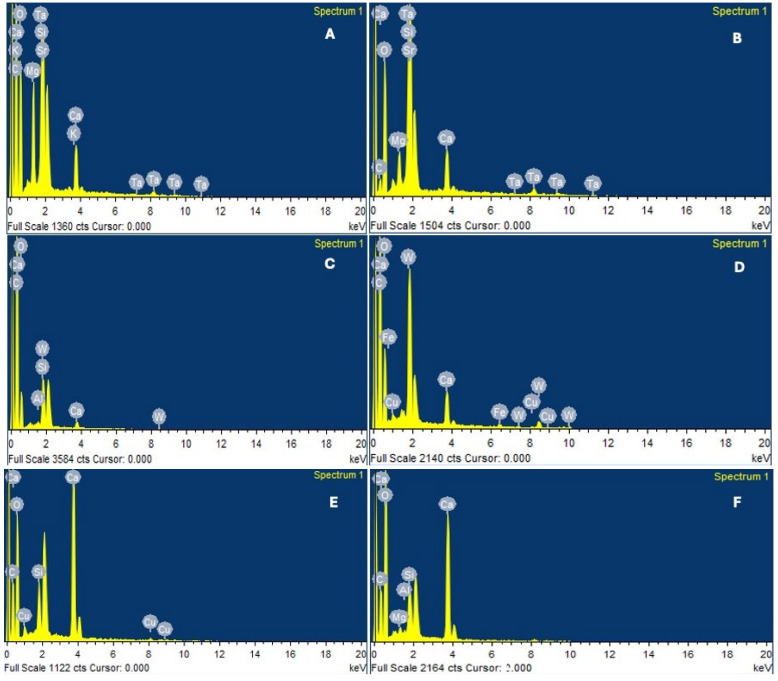
Representative energy-dispersive X-ray spectroscopy spectra of the tested sealers under dry and deionized water conditions after 28 days. (**A**) C-Root SP (dry), (**B**) C-Root SP (deionized water), (**C**) AH Plus (dry), (**D**) AH Plus (deionized water), (**E**) TotalFill BC (dry), and (**F**) TotalFill BC (deionized water). Distinct elemental peaks were observed for each sealer, reflecting differences in composition. C-Root SP exhibited prominent strontium and calcium peaks, with increased relative peak intensity following immersion. AH Plus showed dominant carbon and tungsten peaks, consistent with its epoxy resin matrix and tungsten-based radiopacifier. TotalFill BC displayed characteristic calcium, silicon, and oxygen peaks typical of calcium silicate-based materials, with minor changes observed after immersion.

The elemental composition (wt%) of carbon, oxygen, silicon, calcium, strontium, tungsten, and phosphorus after 28 days under dry and DIW conditions is presented in Table 5 and illustrated in Figure 3. Two-way ANOVA revealed statistically significant effects of sealer type, storage condition (dry vs. DIW), and their interaction on carbon, oxygen, silicon, and calcium concentrations (*p* < 0.001 unless otherwise indicated).

Carbon content decreased after DIW immersion in all sealers, with the lowest value observed in C-Root SP under DIW conditions (9.26 ± 0.03 wt%). Oxygen content increased after DIW immersion across all materials, with TotalFill BC under DIW conditions showing the highest oxygen content (59.11 ± 0.03 wt%).

Calcium content increased following DIW immersion in C-Root SP and AH Plus, whereas TotalFill BC showed a reduction in calcium content under DIW conditions compared with dry storage.

Silicon was not detected in AH Plus under DIW conditions. In C-Root SP, silicon increased from 2.68 ± 0.03 wt% (dry) to 4.49 ± 0.02 wt% (DIW), whereas in TotalFill BC, silicon decreased slightly from 3.61 ± 0.01 wt% (dry) to 3.20 ± 0.01 wt% (DIW).

Strontium was detected exclusively in C-Root SP and increased from 13.67 ± 0.05 wt% (dry) to 29.49 ± 0.06 wt% (DIW). Tungsten was detected only in AH Plus and increased from 12.80 ± 0.04 wt% (dry) to 28.07 ± 0.06 wt% (DIW). Phosphorus was not detected in any group under either storage condition. Independent *t*-tests were used for within-sealer comparisons of sealer-specific elements (strontium and tungsten).

It should be noted that EDX analysis provides surface-sensitive, semi-quantitative elemental information and does not necessarily reflect bulk material composition. Therefore, these findings should be interpreted as indicative of surface compositional changes rather than definitive evidence of underlying physicochemical mechanisms.

### 3.5. SEM Surface Morphology

**Figure 4:**
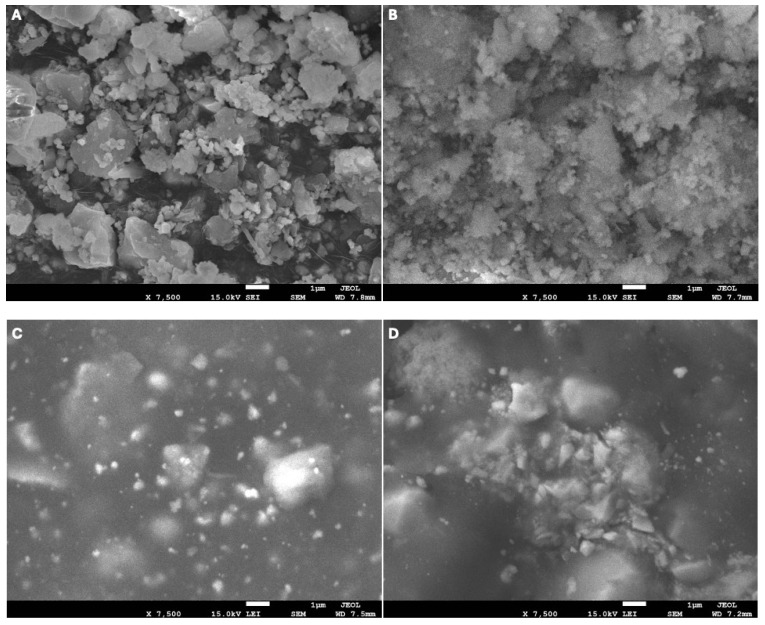

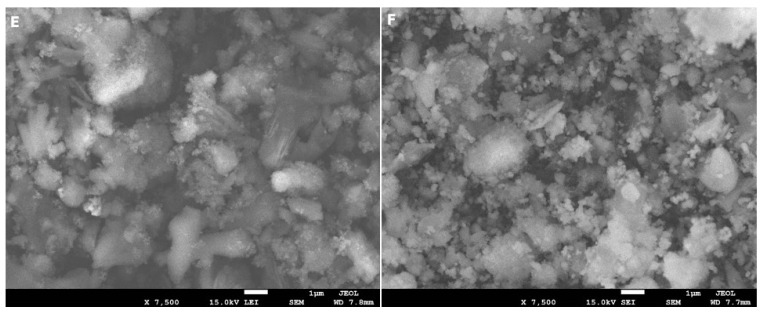
Representative SEM micrographs (7500× magnification) showing surface morphology of the tested sealers after 28 days under dry and deionized water (DIW) conditions: (**A**) C-Root SP (dry), (**B**) C-Root SP (DIW), (**C**) AH Plus (dry), (**D**) AH Plus (DIW), (**E**) TotalFill BC (dry), and (**F**) TotalFill BC (DIW).

Representative SEM micrographs of the tested sealers after 28 days under dry and deionized water (DIW) conditions are presented in Figure 4. Under dry conditions, C-Root SP exhibited a relatively compact surface with fine granular morphology. AH Plus demonstrated a smooth and homogeneous surface with limited surface irregularities. TotalFill BC displayed a heterogeneous surface characterized by crystalline-like features and visible surface porosities. Following 28 days of immersion in DIW, surface alterations were observed in all groups. C-Root SP showed increased surface irregularity with the presence of granular deposits and microporosities. AH Plus exhibited mild surface roughening compared with its dry condition, with no evident crystalline deposition. TotalFill BC demonstrated increased surface coarseness and microporosity, along with clustered surface features distributed across the specimen.

Overall, more noticeable morphological alterations after DIW immersion were observed in C-Root SP and TotalFill BC compared with AH Plus.

## 4. Discussion

The present study evaluated the physicochemical behavior of a novel strontium silicate-based sealer (C-Root SP) in comparison with a premixed calcium silicate-based sealer (TotalFill BC) and an epoxy resin-based sealer (AH Plus). Significant differences were identified among the materials in setting time, net mass change (apparent solubility behavior), alkalinity, and surface composition after 28 days of aging. While conventional calcium silicate and resin-based sealers have been extensively characterized, limited data are available regarding strontium-modified silicate formulations used as root canal sealers. Because strontium incorporation has been reported to influence hydration kinetics, ionic diffusion, and potentially bioactive behavior [36,39,40], the present findings provide important comparative insight into how such compositional modification may affect physicochemical performance. Based on the findings of the present study, the null hypothesis was rejected, as significant differences were observed among the tested sealers across all evaluated physicochemical parameters.

AH Plus exhibited the longest final setting time (37.33 ± 0.13 h), which was approximately 4.6 h longer than C-Root SP (32.75 ± 0.57 h) and 7.1 h longer than TotalFill BC (30.18 ± 0.20 h), representing increases of approximately 14% and 23%, respectively. The initial setting time of AH Plus (10.93 ± 0.65 h) is consistent with values reported by Zhou et al. [20], who documented an initial setting time of 11.5 ± 1.5 h under ISO conditions. Loushine et al. [19] reported shorter setting times for premixed bioceramic sealers compared with resin-based materials. The present findings therefore support previous evidence that epoxy resin-based sealers typically exhibit longer setting times than calcium silicate-based materials.

The differences observed among materials are consistent with their distinct setting mechanisms. AH Plus undergoes a polymerization reaction between epoxy resins and amine hardeners, as described by Ørstavik et al. [8], a process that progresses gradually and may be influenced by environmental factors. In contrast, bioceramic sealers set through hydration of calcium silicate phases, forming calcium silicate hydrate (C-S-H) gel and calcium hydroxide, as previously reported [19,41]. Hydration-driven reactions promote progressive matrix stabilization once sufficient moisture is available, which may explain the comparatively shorter setting times observed for TotalFill BC and C-Root SP.

Strontium incorporation into calcium silicate matrices may further influence hydration kinetics. Partial substitution of calcium with strontium within the silicate lattice has been shown to modify phase reactivity and dissolution dynamics [39,40]. Experimental studies on Sr-doped calcium silicate systems demonstrated alterations in early physicochemical performance and matrix development [39,40]. In the present study, the intermediate setting time of C-Root SP suggests that strontium substitution may modulate hydration behavior without markedly accelerating or delaying matrix formation relative to conventional calcium silicate formulations.

Although ISO 6876 specifies a specimen thickness of 2 mm, a 3 mm thickness was used to ensure adequate bulk material and reduce potential edge-related desiccation. This represents a methodological modification of the ISO protocol and should be interpreted as an adapted approach rather than strict compliance. Similar methodological adjustments have been reported in previous physicochemical investigations evaluating calcium silicate-based sealers [32,36,42].

C-Root SP exhibited net mass change values (net mass loss) throughout the evaluation period, increasing from 5.32 ± 4.72% at 24 h to 6.83 ± 5.55% at 28 days, whereas TotalFill BC demonstrated negative values indicative of net mass gain (−4.28 ± 0.64% to −2.69 ± 0.54%), and AH Plus showed minimal dimensional change (−0.15 ± 0.21% to −0.60 ± 0.23%). These values represent overall mass variation rather than true solubility, as negative values likely reflect water sorption and/or surface precipitation rather than material dissolution. Although C-Root SP net mass change values exceeded the ISO 3% threshold, the absence of a consistent progressive increase over time suggests stabilization following initial hydration-related ion release.

Similar hydration-driven mass changes have been reported for calcium silicate-based sealers [6,32,43,44]. Elyassi et al. [11] demonstrated measurable calcium-containing leachates during early immersion, while Urban et al. [32] reported 14-day solubility values of approximately 1.60% for BioRoot RCS and 3.38% for MTA Fillapex, compared with ≤0.54% for AH Plus. The minimal mass change observed for AH Plus in the present study is consistent with its polymerization-based chemistry and limited ion exchange capacity [7].

The negative values observed for TotalFill BC likely reflect net water sorption and surface precipitation phenomena during ongoing hydration [43,44]. Previous investigations have shown that the storage medium influences apparent solubility, with phosphate-containing solutions promoting hydroxyapatite deposition that partially offsets material dissolution [32]. Although deionized water was used in the present study, SEM findings demonstrated surface deposition after immersion, suggesting a dynamic balance between ion release and surface mineral formation processes.

The relatively high standard deviation observed for C-Root SP reflects heterogeneous mass change behavior, likely due to the simultaneous occurrence of dissolution and hydration-related surface precipitation. This dual mechanism may result in both mass loss and mass gain among specimens, contributing to increased variability compared with the more uniform behavior of AH Plus and TotalFill BC.

C-Root SP demonstrated markedly higher early apparent alkalinity (14.12 ± 0.05 at 24 h; 14.85 ± 0.02 at 7 days) compared with TotalFill BC and AH Plus. Although pH values declined over time, C-Root SP maintained an alkaline environment at 28 days (10.88 ± 0.10). This behavior is consistent with hydration-driven hydroxyl ion release characteristic of calcium silicate-based materials, which contributes to the observed apparent alkalinity [41,45]. In contrast, TotalFill BC exhibited moderate and relatively stable apparent alkalinity throughout the evaluation period, whereas AH Plus showed lower pH values with a progressive decline over time. Previous studies have reported early pH values typically ranging between 10 and 12 for hydraulic calcium silicate-based sealers [21,32], while epoxy resin-based sealers generally demonstrate lower alkalinity [46].

It should be noted that the same storage medium was maintained throughout the experimental period; therefore, the reported pH values reflect cumulative alkalinization rather than measurements obtained from refreshed solutions. Accordingly, the reported pH values should be interpreted as reflecting cumulative ion release within a closed system rather than discrete time-point alkalinity under refreshed conditions.

Strontium incorporation may further contribute to enhanced early ion diffusion. Wu et al. [39] and Abdalla et al. [36] reported that strontium substitution alters the silicate lattice structure and increases ionic mobility, potentially facilitating hydroxyl ion release during early hydration. From a biological perspective, McHugh et al. [47] demonstrated that elimination of Enterococcus faecalis requires pH values above 11, suggesting that the cumulative alkalinization observed for C-Root SP may be relevant to antimicrobial effects under alkaline conditions, although this was not directly evaluated in the present study.

The pH values recorded for C-Root SP exceeded 14 at early time points and should be interpreted with caution. Such values likely reflect limitations of conventional glass electrode measurements under highly alkaline conditions and elevated ionic strength rather than absolute pH values. Reduced accuracy of pH electrodes in extreme alkaline environments due to alkali error and sensor instability has been previously reported [48].

A strong positive correlation between net mass change (apparent solubility behavior) and pH was observed for C-Root SP (r = 0.86; R^2^ = 0.75), suggesting an association between ion diffusion–related mass change and alkalinity. In contrast, the negative correlation observed for AH Plus reflects its non-hydraulic, polymerization-based mechanism, which limits ion exchange and hydroxyl ion release. These associations should be interpreted as descriptive rather than indicative of direct causative relationships and are consistent with previous reports demonstrating an association between sustained alkalinity and net mass change behavior in calcium silicate-based sealers [32].

SEM-EDX analysis demonstrated hydration-related surface and compositional changes following aqueous aging. The observed decrease in carbon and increase in oxygen signals after immersion are consistent with previously reported hydration-associated changes in calcium silicate-based materials [49]. Redistribution of calcium and silicon may indicate ongoing ion exchange and surface maturation processes [43].

The enrichment of strontium detected in C-Root SP after immersion is consistent with previous observations that strontium-modified silicate systems exhibit altered surface reactivity and nucleation behavior [27,36]. Although apatite formation typically requires phosphate-containing media [50], the calcium- and silicon-rich surface characteristics observed under deionized water conditions may reflect early-stage hydration and surface transformation processes rather than definitive evidence of biomineralization [42]. Given that EDX provides surface-sensitive and semi-quantitative analysis, these findings should be interpreted with caution and do not necessarily reflect bulk material composition or confirm underlying physicochemical mechanisms.

The use of deionized water represents a limitation, as it does not replicate phosphate-rich physiological conditions required for complete assessment of apatite formation. However, deionized water was intentionally used in the present study to isolate intrinsic hydration-driven physicochemical behavior, including ion release, alkalinity, and net mass change, without the confounding influence of externally supplied calcium and phosphate ions. In contrast, simulated body fluid (SBF), as described by Kokubo and Takadama [51], provides an ionically balanced environment that promotes calcium phosphate and apatite precipitation, thereby better simulating in vivo conditions. Under such conditions, the interaction between released calcium ions and phosphate ions may alter apparent net mass change, pH evolution, and surface composition through precipitation processes that partially offset material dissolution. Consequently, values obtained in SBF may differ from those observed in deionized water, particularly for calcium silicate-based sealers that exhibit bioactive behavior.

Therefore, the present findings should be interpreted as reflecting intrinsic material properties under simplified aqueous conditions rather than demonstrating definitive bioactivity under physiological conditions. Furthermore, in vitro conditions cannot fully reproduce clinical variables such as dentin moisture, mechanical loading, and thermal fluctuations. In addition, the present study evaluated selected short-term physicochemical properties only, while other clinically relevant parameters such as sealing ability, mechanical properties, long-term dimensional stability, and biological response were not assessed.

Within the limitations of this in vitro investigation, C-Root SP demonstrated physicochemical behavior comparable to established hydraulic sealers, with enhanced early apparent alkalinity and hydration-related surface changes that may be associated with strontium incorporation. The differences observed among materials are likely attributable to compositional variations within calcium silicate systems rather than definitive functional superiority.

However, the clinical relevance of these findings cannot be directly inferred from the present in vitro data. Further studies under simulated physiological conditions and in vivo models are required to better evaluate the functional performance and clinical applicability of strontium-modified calcium silicate sealers.

## 5. Conclusions

Within the limitations of this in vitro investigation, C-Root SP demonstrated physicochemical characteristics consistent with hydration-driven calcium silicate-based sealers, including sustained apparent alkalinity, ion exchange-associated behavior, and surface compositional changes following aqueous aging. Strontium incorporation may influence hydration-related physicochemical behavior without clear evidence of improved material stability compared with established hydraulic sealers.

These findings provide foundational evidence on the physicochemical profile of strontium silicate-based endodontic sealers and should be interpreted within the inherent limitations of an in vitro study.

### Clinical Significance

Strontium-enriched C-Root SP demonstrated physicochemical characteristics consistent with hydration–driven calcium silicate sealers, including sustained apparent alkalinity and surface compositional changes. While these properties may influence sealer–dentin interaction and interfacial behavior under hydrated conditions, further in vitro and in vivo investigations are required to determine their clinical relevance.

## Figures and Tables

**Table 1:** Initial and Final Setting Times (h) of the Tested Sealers (mean ± SD).

Test	AH Plus (h)	C-Root SP (h)	TotalFill BC (h)	*p*-Value
Initial Setting Time	10.93 ± 0.65 ^a^	9.35 ± 0.38 ^b^	7.98 ± 0.32 ^c^	<0.001
Final Setting Time	37.33 ± 0.13 ^a^	32.75 ± 0.57 ^b^	30.18 ± 0.20 ^c^	<0.001

Values are presented as mean ± standard deviation. Different lowercase superscript letters within the same row indicate statistically significant differences among sealers (one-way ANOVA followed by Tukey’s post hoc test, *p* < 0.05).

**Table 2:** Net Mass Change (apparent solubility behavior) of Tested Sealers Across Time Intervals (mean ± SD).

Time	AH Plus %	C-Root SP %	TotalFill BC %	*p*-Value
24 h	−0.15 ± 0.21 ^a^	5.32 ± 4.72 ^b^	−4.28 ± 0.64 ^c^	<0.001
7 d	−0.25 ± 0.29 ^a^	2.28 ± 4.61 ^b^	−2.20 ± 0.62 ^c^	<0.001
14 d	−0.78 ± 0.20 ^a^	3.35 ± 4.27 ^b^	−3.79 ± 0.56 ^c^	<0.001
21 d	−0.56 ± 0.20 ^a^	5.61 ± 6.38 ^b^	−2.65 ± 0.56 ^c^	<0.001
28 d	−0.60 ± 0.23 ^a^	6.83 ± 5.55 ^b^	−2.69 ± 0.54 ^c^	<0.001

Values are presented as mean ± standard deviation. Different lowercase superscript letters within the same row indicate statistically significant differences among sealers (one-way ANOVA followed by Tukey’s post hoc test, *p* < 0.05). *p*-values for within-sealer comparisons over time were obtained using repeated-measures ANOVA: AH Plus (*p* = 0.31), C-Root SP (*p* = 0.30), and TotalFill BC (*p* = 0.005). Negative values indicate apparent mass gain, whereas positive values indicate net mass loss.

**Table 3:** pH Values of Tested Sealers at Different Time Intervals (mean ± SD).

Time	AH Plus	C-Root SP	TotalFill BC	*p*-Value
24 h	9.68 ± 0.25 ^b^^A^	14.12 ± 0.05 ^a^^A^	10.95 ± 0.08 ^c^^A^	<0.001
7 d	10.04 ± 0.14 ^c^^A^	14.85 ± 0.02 ^a^^A^	11.09 ± 0.21 ^b^^A^	<0.001
14 d	10.82 ± 0.40 ^c^^A^	14.11 ± 0.32 ^a^^A^	11.44 ± 0.23 ^b^^A^	<0.001
21 d	7.01 ± 0.61 ^c^^A^	12.88 ± 0.37 ^a^^A^	11.00 ± 0.16 ^b^^A^	<0.001
28 d	6.23 ± 0.09 ^c^^B^	10.88 ± 0.10 ^a^^B^	10.67 ± 0.21 ^b^^B^	<0.001

Values are presented as mean ± standard deviation. Different lowercase letters (a–c) within the same row indicate statistically significant differences among sealers at the same time point (one-way ANOVA followed by Tukey’s post hoc test, *p* < 0.05). Different uppercase letters (A, B) within the same column indicate statistically significant differences within each sealer over time (repeated-measures ANOVA, *p* < 0.05). Values exceeding pH 14 reflect measurement limitations under highly alkaline conditions and should be interpreted as indicative of strong alkalinity rather than absolute pH values.

**Table 4:** Pearson Correlation and Linear Regression between Net mass change (apparent solubility behavior) and pH (28-Day Evaluation Period).

Sealer	Pearson Correlation (r)	Direction of Association	R^2^ (Regression)
AH Plus	−0.79	Negative	0.62
C-Root SP	+0.86	Positive	0.75
TotalFill BC	−0.58	Negative	0.34

**Table 5:** Elemental composition (wt%) determined by EDX after 28 days under dry and DIW conditions.

Element	C-Root Dry	C-Root DIW	AH Plus Dry	AH Plus DIW	TotalFill Dry	TotalFill DIW	*p*-Value
C	45.02 ± 0.14 ^b^	9.26 ± 0.03 ^d^	62.67 ± 0.05 ^a^	43.12 ± 0.06 ^b^	16.69 ± 0.03 ^c^	14.66 ± 0.02 ^c^	<0.001
O	23.86 ± 0.13 ^c^	35.70 ± 0.08 ^b^	20.73 ± 0.05 ^c^	21.78 ± 0.02 ^c^	49.34 ± 0.04 ^a^	59.11 ± 0.03 ^a^	<0.001
Si	2.68 ± 0.03 ^c^	4.49 ± 0.02 ^b^	1.93 ± 0.01 ^c^	-	3.61 ± 0.01 ^b^	3.20 ± 0.01 ^b^	<0.01 *
Ca	3.85 ± 0.04 ^c^	6.40 ± 0.02 ^b^	1.54 ± 0.01 ^d^	4.66 ± 0.01 ^c^	27.87 ± 0.02 ^a^	22.09 ± 0.02 ^a^	<0.001
Sr	13.67 ± 0.05 ^b^	29.49 ± 0.06 ^a^	-	-	-	-	<0.001 †
W	-	-	12.80 ± 0.04 ^b^	28.07 ± 0.06 ^a^	-	-	<0.001 †
P	ND	ND	ND	ND	ND	ND	-

Values are presented as mean ± standard deviation. Different lowercase superscript letters within the same row indicate statistically significant differences among groups (*p* < 0.05). *p*-values for carbon, oxygen, silicon, and calcium were obtained using two-way ANOVA (sealer × storage condition), while *p*-values for sealer-specific elements (strontium and tungsten) were obtained using independent *t*-tests comparing dry and DIW conditions within each material. ND = not detected; “-” = not applicable. “*” indicates analysis limited to applicable materials; “†” indicates comparison performed within a single material only.

## Data Availability

The original contributions presented in the study are included in the article, further inquiries can be directed to the corresponding author.
